# The impact of war on the development and progression of arterial hypertension and cardiovascular disease: protocol of a prospective study among Ukrainian female refugees

**DOI:** 10.3389/fcvm.2023.1324367

**Published:** 2024-01-11

**Authors:** A. Shalimova, M. S. Stoenoiu, W. J. Cubała, M. Burnier, A. Persu, K. Narkiewicz

**Affiliations:** ^1^ Department of Hypertension and Diabetology, Medical University of Gdańsk, Gdańsk, Poland; ^2^ Department of Internal Medicine, Rheumatology, Cliniques Universitaires Saint-Luc, Université Catholique de Louvain, Brussels, Belgium; ^3^ Institut de Recherche Expérimentale et Clinique, Université Catholique de Louvain, Brussels, Belgium; ^4^ Department of Psychiatry, Faculty of Medicine, Medical University of Gdańsk, Gdańsk, Poland; ^5^ Faculty of Biology and Medicine, University of Lausanne, Lausanne, Switzerland; ^6^ Division of Cardiology, Cliniques Universitaires Saint-Luc, Université Catholique de Louvain, Brussels, Belgium; ^7^ Pole of Cardiovascular Research, Institut de Recherche Expérimentale et Clinique, Université Catholique de Louvain, Brussels, Belgium

**Keywords:** stress, post-traumatic stress disorder, hypertension, refugees, cardiovascular disease

## Abstract

**Background:**

Growing evidence supports the impact of psychological factors such as traumatic experiences and Post Traumatic Stress Disorder (PTSD) on the incidence of arterial hypertension (HTN) and cardiovascular diseases (CVD). The war in Ukraine is exposing million inhabitants to traumatic experiences and severe stress. Part of Ukrainians (mostly women and children) left the country to escape war. We report the protocol of a prospective study aiming at the assessment of the impact of war-induced stress on HTN and CVD in women Ukrainian refugees who moved to Poland.

**Methods and design:**

The study will be conducted in 3 stages. Stage 1 will assess the prevalence of HTN and PTSD among Ukrainian refugees and will estimate the impact of war-related trauma exposure on these parameters. Data on office blood pressure (BP) will be compared to data already collected in STEPS data 2019 and May Measurement Month 2021 in Ukraine, matched for age and sex. Stage 2 will involve subjects diagnosed with HTN and/or PTSD referred for management and follow-up of these conditions. Psychologic targeted therapies will be offered to subjects with confirmed PTSD, with a periodical reassessment of the severity of PTSD-associated symptoms and of its impact on HTN and cardiovascular health. Clinical history and characteristics will be compared among three groups: subjects with HTN and PTSD, with HTN without PTSD, with PTSD but without HTN. Stage 3 will involve a subgroup among those screened in Stage 1, with the objective of investigating the biological mechanisms underlying the relation between HTN and trauma exposure, identifying early signs of subclinical target organ damage in subjects with HTN with/without PTSD.

**Discussion:**

This study will test the hypothesis that trauma exposure and psychological stress contribute to BP elevation and progression of CVD in this population. It will provide new evidence on the effect of an integrated management, including psychological therapy, on BP and cardiovascular risk. Such approach may be further tested and extrapolated to other populations exposed to war and chronic violence, migrants and refugees around the world.

**Research Study Registration:**

number 2022/45/P/NZ5/02812.

## Introduction

Arterial Hypertension (HTN) is the leading cause of cardiovascular morbidity and mortality worldwide (1). High sodium dietary intake, excessive alcohol consumption, obesity and physical inactivity are well-established modifiable risk factors for development of HTN and cardiovascular diseases (2). Nevertheless, growing evidence supports the impact of psychological factors on HTN development and maintenance, in particular coping, emotion expression/repression regulation, traumatic experiences, and Post Traumatic Stress Disorder (PTSD) (3–6).

In particular, PTSD has been associated with the development of hypertension and cardiovascular diseases. Underlying mechanisms may include both physiological mechanisms such as activation of autonomous system, renin angiotensin system and inflammation, and behavioural mechanisms, such as an unhealthy lifestyle and poor drug adherence (7, 8).

Data from a large 20-year study of nearly 50,000 American nurses found that exposure to trauma and increased symptoms of PTSD may increase the risk of cardiovascular disease (CVD) in this group of women (9). Although there is growing evidence linking PTSD to major CVD risk factors and major CVD outcomes, it remains unclear whether these associations are causal or confounding (10). Research in this area has shown that treatments aimed at reducing the risk of CVD need to address both the diagnostic components of PTSD and the associated risk factors for CVD (11).

Moreover, the mechanisms underlying these associations have primarily been studied in specific populations such as soldiers, and more information is needed to assess whether these early observations are applicable to other patient populations and different types of traumatic experience, for example in refugees.

The association between PTSD and hypertension has been particularly well documented in military servants and veterans (12–14), and more recently in subjects exposed to war and chronic violence (6, 15) and in patients with resistant hypertension (16, 17). Among the latter a substantial number proved to have a history of severe trauma or were migrants/refugees previously exposed to mass violence (18). The prevalence of PTSD in refugees is extremely high, usually in the range of 20%–40% (19) compared to an estimate lifetime prevalence of PTSD ∼6% in the US population (20). The current war in Ukraine is causing exposure of millions of inhabitants to traumatic experiences and severe stress. Part of Ukrainian population left the country to escape war. As of now, approximately 2 million Ukrainian refugees (mostly women and children) are present in Poland (21).

However, to our knowledge, the association of HTN with PTSD in refugees from Ukraine and more generally from regions exposed to war has not been investigated. Furthermore, few data are currently available about the clinical and socio-demographical determinants of the association between HTN and PTSD. In particular, despite the fact that the prevalence of PTSD is higher in women than in men (9, 20), as most evidence is derived from war veterans, the relation between PTSD and hypertension is poorly described in women. More generally, it is not clear why and who is at higher risk of developing HTN and adverse cardiovascular outcomes among people exposed to traumatic experiences. One does not know whether the outcome depends more on the type and intensity of the trauma or on individual responses to it or on both. Furthermore, even if previous studies support the independent role of PTSD *per se* in the development of HTN and cardiovascular outcomes, the concomitant interaction with other unhealthy lifestyle habits is often poorly defined.

The pathophysiology of HTN in response to trauma exposure remains poorly investigated. Previous hypotheses included involvement of immune system and inflammatory response, and activation of sympathetic and renin-angiotensin systems (14). A better understanding of underlying mechanisms may improve the management of stress-induced hypertension in the future, especially for patients with drug-resistant or refractory HTN associated with PTSD.

Finally, while it is likely that timely psychological management of PTSD may change HTN natural history and reduce cardiovascular disease burden no previous study has analysed the potential effect of PTSD-targeted therapy on HTN and cardiovascular outcomes occurrence.

Thus, the current study aims to address these gaps with a three stages approach: stage 1 will assess the prevalence of PTSD and HTN among female Ukrainian refugees, stage 2 will evaluate the effect of early targeted-PTSD therapy on cardiovascular risk profile and stage 3 will identify early signs of subclinical target organ damage in subjects with HTN with or without PTSD and characterize the biological pathways potentially involved in the relation between HTN and PTSD (Stage 3).

## Methods and analysis

The protocol of this study was approved by the Bioethics Committee for Scientific Research at the Medical University of Gdansk, Poland (reference number: NKBBN/558/2022). Written informed consent will be obtained from each participant. All procedures will be conducted in accordance with the Declaration of Helsinki.

### Study design

**Figure 1:**
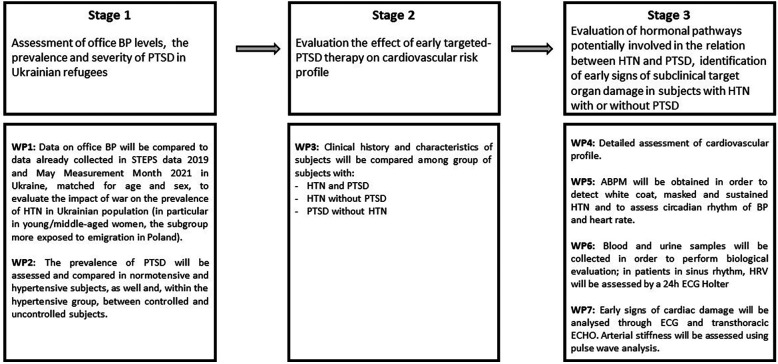
Stages and work packages of the study.

**Figure 2:**
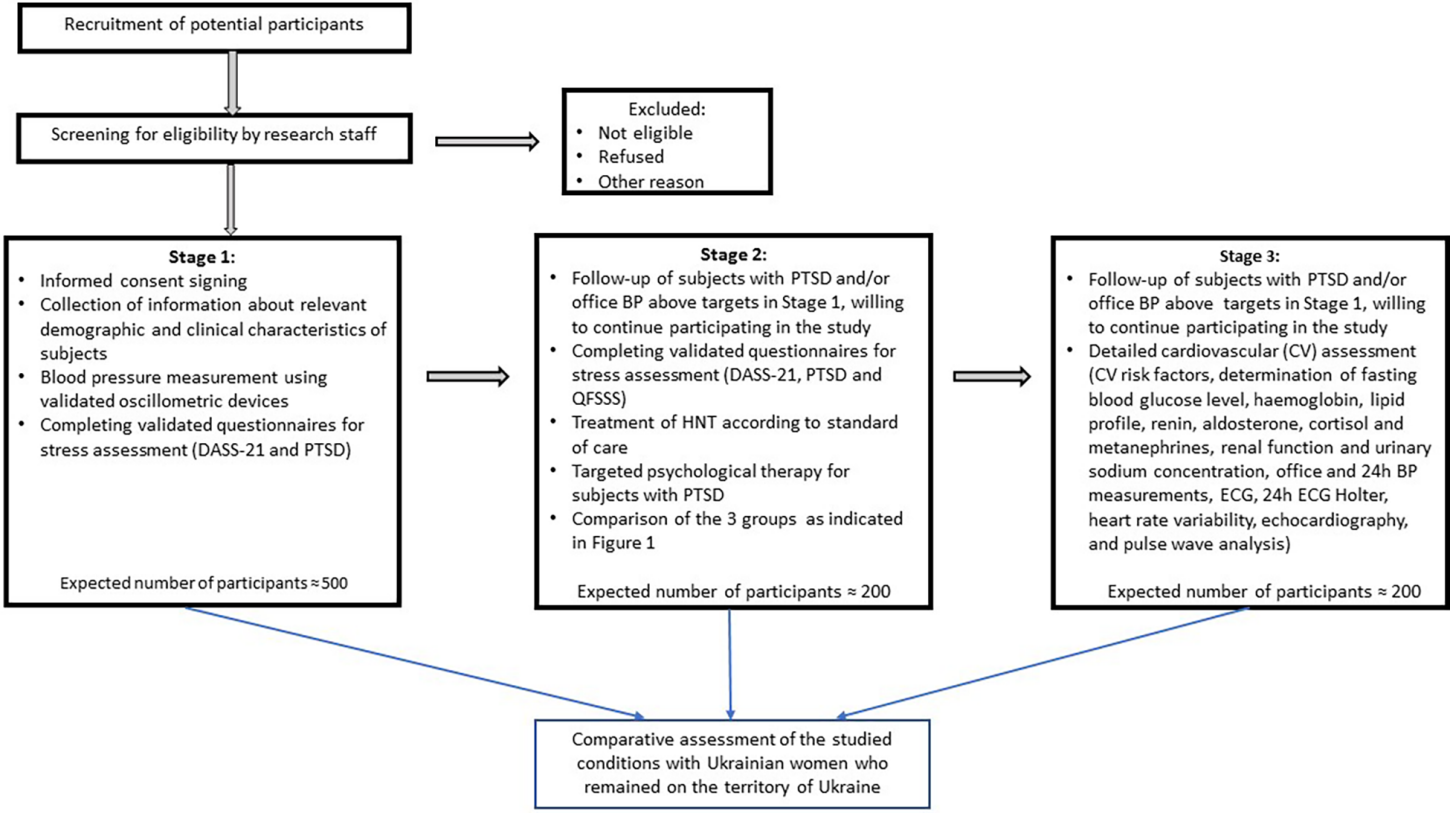
Study design.

This research will be performed at the Division of Hypertension and Diabetology of the Medical University of Gdansk, Poland. The general work plan of this research includes 3 stages and 7 work packages (WPs) (Figures 1, 2).

The objective of *Stage 1* is to assess the prevalence and severity of PTSD, as well as the levels of office blood pressure (BP) in Ukrainian refugees willing to participate to this initial screening stage. The distribution of BP and the prevalence of HTN (defined as a systolic BP > 140, diastolic BP > 90 mmHg and/or being on antihypertensive treatment) will be compared to cross-sectional data available from STEPS study (National study on the prevalence of major risk factors for noncommunicable diseases, in line with the WHO-approved phased approach to surveillance) (22) and from May Measurement Month 2021 in Ukraine, matched for age and sex (WP1). Information on previous and current antihypertensive therapies will be collected.

The prevalence of PTSD will be compared between normotensive and hypertensive subjects, as well and, within the hypertensive group, between controlled and uncontrolled/drug-resistant subjects (WP2).

*Stage 2* will involve the follow-up of patients who received a diagnosis of PTSD and/or with office BP above reference values in Stage 1, willing to participate also to this study stage. HTN will be managed and treated following standard of care as recommended by guidelines of the European Society of Hypertension (23). Patients with a diagnosis of PTSD will benefit from targeted psychological therapies and the diagnosis of PTSD and its severity will be periodically reassessed.

Clinical history and characteristics of subjects will be compared among group of subjects with HTN and PTSD, with HTN and without PTSD and with PTSD but without HTN (WP3).

The aim of *Stage 3* is to study in more details the mechanisms underlying the association between HTN and PTSD, by comparing patients with HTN, PTSD, both or none of these two diagnoses. This study stage will involve a subgroup of subjects, screened in Stage 1 and willing to participate to this study stage.

In these subjects, main cardiovascular risk factors other than HTN will be evaluated. Assessment will include smoking, alcohol consumption, physical activity, and dietary habits, as well as the determination of fasting blood glucose level, glycated haemoglobin, lipid profile, renal function and urinary sodium concentration (WP4). A 24 h ambulatory BP measurement (ABPM) will be obtained in order to detect white coat, masked and sustained HTN and to assess circadian rhythm of BP (WP5). Renin-angiotensin system and adrenal function will be evaluated on the basis of blood and urine samples. Furthermore, sympathetic nerve activity will be assessed by heart rate variability (HRV) (WP6). An evaluation of early signs of target organ damage will be performed using electrocardiogram, transthoracic echocardiography, and pulse wave analysis (WP7).

In summary, the specific research goals for each stage of the project will be the following:

*Stage 1*: To assess the prevalence of PTSD and HTN among Ukrainian refugees. To evaluate the impact of war-induced trauma on HTN prevalence, by comparison with available data of previous large-scale epidemiological data. To evaluate the socio-demographical factors mediating the association between PTSD and HTN by comparison of normotensive and hypertensive subjects and controlled vs. uncontrolled/resistant hypertensive subjects.

*Stage 2*: To evaluate the effect of early targeted-PTSD therapy on cardiovascular risk profile. To compare the natural history, treatment response and target organ damage evolution in hypertensive patients with and without PTSD.

*Stage 3*: To characterize hormonal pathways potentially involved in the relation between HTN and PTSD. To identify early signs of subclinical target organ damage in subjects with HTN with or without PTSD.

The different research stages will be analysed in order to detect potential hazards and assess risk level, and mitigation measures will be applied if the risk level is unacceptable.

### Criteria for inclusion/exclusion from the study

Our study will include women over the age of 18 who came from Ukraine to Poland after February 24, 2022 and at the time of their inclusion in the study living in the Tricity (Gdańsk, Sopot, Gdynia).

In order for the sample to be as representative as possible and fully reflect information about forced migrants from Ukraine to Poland, in our study there is no upper age limit and no restrictions on any concomitant diseases. Inclusion of patients in the study will be determined based on their willingness to participate in the project, an arrival in Poland after February 24, 2022, female gender, age of 18 years and older, actual location in the Tricity and signed informed consent.

### Recruitment

Participants will be recruited through promotion of this project in Sopot Centre for Integration and Support for Foreigners. A written informed consent in Ukrainian language will be obtained from all participants after the nature of the study is explained by an Ukrainian-speaking cardiologist. Participation will only be possible after written informed consent is provided.

### Research methodology

*Stage 1* of the project will be organized in form of screening campaigns among Ukrainian refugees. Promotion of these screening campaigns will take place in Sopot Centre for Integration and Support for Foreigners. Patients will be examined in an ambulatory environment by Ukrainian- speaking investigators, thus providing them the possibility to access the Polish health system speaking in their native language. Short past medical history information will be collected at this stage, including known history of HTN, known HTN-associated organ damage, concomitant cardiovascular risk factors (i.e., smoking status, diabetes, renal disease, dyslipidaemia) and comorbidities, previous cardiovascular or cerebrovascular events, and past and current therapy.

Office BP will be measured in seated position in a quiet environment, for three consecutive readings in both arms, using an oscillometric validated device Microlife WatchBP office ABI (24). Two further measurements will be performed after 1 and 3 min in standing position.

We will collect data about relevant demographic and clinical characteristics of subjects included in the study.

To assess general emotional distress, we will use validated questionnaire DASS-21 (Depression, Anxiety and Stress Scale—21). On the basis of this questionnaire, we may determine 5 levels of severity of the depression, stress and anxiety: normal, mild, moderate, severe and extremely severe (25, 26).

PTSD diagnosis according to DSM-5 will be based on the presence of the following criteria: exposure to actual or threatened death, serious injury, or sexual violence by direct experiencing, witnessing in person, learning the occurrence in a close family member or friend, or repeated or extreme exposure to details about the traumatic event; presence of one or more intrusion symptom, expressed as intrusive memories, recurrent dreams, dissociative reactions, and intense psychological distress or reactions related to the traumatic event; one or more avoidance symptom, expression of persistent avoidance or effort to avoid internal and/or external stimuli associated with the traumatic event; two or more negative alterations in cognitions and mood; two or more alterations in arousal and reactivity; disturbance duration longer than 1 month; clinically significant distress or impairment in social, occupational, or other important areas of functioning; disturbance not attributable to the effects of a medication, substance or another medical condition (27).

Assessment of PTSD and trauma exposure will be performed using the PCL-5 questionnaire, designed for self-administration and for PTSD screening and severity assessment (28). On the basis of this questionnaire, a provisional PTSD diagnosis will be obtained in presence of at least 1 PTSD symptom in B item (questions 1–5), 1 in C item (questions 6–7), 2 in D items (questions 8–14), 2 in E items (questions 15–20). The presence of each symptom will be defined by a rate of 2 or more (“moderately” or “higher”) (28).

Data on office BP will be compared to data already collected in STEPS data 2019 (national study on the prevalence of major risk factors for noncommunicable diseases, in line with the WHO-approved phased approach to surveillance) (22) and May Measurement Month 2021 in Ukraine, matched for age and sex, in order to evaluate the impact of war on prevalence of HTN in Ukrainian population (in particular in young/middle-aged women, the subgroup more exposed to emigration in Poland) (WP1). In particular, we will compare the demographics of our sample of Ukrainian refugee women with the female sample from STEPS 2019 and May Measurement Month 2021 in Ukraine. If significant differences arise between our sample and the comparison population in specified studies, we will perform additional adjustments and analyses.

Based on office BP, subjects will be classified in normotensive and hypertensive, according to ESH guidelines (23), and in the latter category, subjects will be classified in newly diagnosed hypertensive, controlled known hypertensive, uncontrolled known hypertensive, and resistant hypertensive patients, also based on medical history (known history of hypertension, number of antihypertensive drugs) (WP2). PTSD prevalence and severity will be compared among these groups.

*Stage 2* will involve the follow-up of patients who will have been diagnosed with PTSD and/or with office BP above reference values in Stage 1, also willing to participate to this study stage. Information about lifestyle habits, such as smoking status, alcohol consumption, physical activity and dietary habits will be integrated/collected from data already gathered at Stage 1. HTN will be managed and treated following standard of care. Subjects will be periodically reassessed to evaluate BP control. PTSD diagnosis will be confirmed on the basis of more advanced evaluation, i.e., Clinician-Administered PTSD Scale for DSM-5 (CAPS-5) (28). Patients will re-complete the stress assessment questionnaires from the Stage 1 of the study (DASS-21 and PTSD-questionnaire), and in addition to the previous two questionnaires, they will complete a questionnaire assessing satisfaction with social support—the Questionnaire on the Frequency of and Satisfaction with Social Support (QFSSS) (29).

Patients with a confirmed diagnosis of PTSD will be referred to a psychiatrist or psychologist, and followed according to standard of care and periodically reassessed for the severity of PTSD, using PCL-5 Questionnaire. Clinical history and characteristics of subjects will be compared among groups of subjects with HTN and PTSD, with HTN and without PTSD, and with PTSD but without HTN (WP3). Furthermore, longitudinal data of subjects with HTN and PTSD will be analysed, in order to evaluate changes in BP and PTSD severity, also in response to psychological and antihypertensive treatment.

For *Stage 3* of the project, voluntary subjects with HTN, PTSD, both or none of these two diagnoses will be selected for further evaluation of mechanisms mediating the association between HTN H and PTSD. Information about lifestyle habits, such as smoking status, alcohol consumption, physical activity and dietary habits will be integrated/collected from data already gathered at Stage 1. Blood and urine samples will be collected in order to screen for other cardiovascular risk factors, such as dyslipidaemia, fasting glucose intolerance and diabetes, renal dysfunction, as well as to quantify sodium dietary intake (WP4).

A 24 h ABPM will be performed as out-of-office measurement technique (WP5). It will allow evaluation of BP circadian rhythm, including dipping status, nocturnal hypertension and increased BP morning rise, as well as classification of subjects in hypertensive, white-coat hypertensive and masked hypertensive. To accurately identify different BP phenotypes (in particular, white coat hypertension, masked hypertension and masked uncontrolled hypertension), ambulatory BP monitoring will be performed on the same day as office BP assessment. White coat HTN in untreated patients will be defined as an office BP in the hypertensive range (≥140/90 mmHg) but 24 h ambulatory and home BP measurements in the normotensive range. Masked HTN will be considered when office BP is in the normotensive range but ambulatory and/or home BP measurements are in the hypertensive range. Masked uncontrolled HTN will be determined in patients receiving antihypertensive therapy with target office BP levels combined with insufficient control of out-of-office BP (23).

Blood and urine samples will also be collected to perform hormonal evaluation: renin, aldosterone, metanephrines, cortisol, C-reactive protein levels will be determined. Furthermore, in patients in sinus rhythm, heart rate variability will be assessed by a 24 h ECG Holter, with inclusion of a 20-min rest period in controlled conditions in ambulatory setting (WP6).

HRV analysis will be performed through time-domain, frequency-domain, and persistent homology methods (30). Early signs of cardiac damage will be analysed by electrocardiogram and transthoracic echocardiography. Sokolow-Lyon and Cornell indexes will be calculated. Echocardiographic assessment will include evaluation of left ventricular geometry and mass, left ventricular systolic and diastolic function, left atrial dimension and wall strain. Systolic and diastolic function will be compared between hypertensive subjects with and without PTSD. Arterial stiffness will be assessed with pulse wave analysis with a SphygmoCor® device. Pulse wave time delay between signals at carotid and femoral site will be used to assess pulse wave velocity (PWV) (WP7).

At each stage of the study data will be collected in pseudo-anonymized form, associated to a code number without any reference to initial, birth date or other personal data of the subject. Only authorized personnel will be able to connect the code number to the identity of the subject. At every stage of the project, subjects will be enrolled only after expression of informed consent, in signed written form. Each participant will be free to leave the study at any moment, without the obligation to provide explanation.

### Statistical analysis

Statistical analysis will be performed using IBM SPSS Statistics Software v28.0.1.1 for Windows (IBM Corp. Released 2021. IBM SPSS Statistics for Windows, Version 28.0. Armonk, NY: IBM Corp). Continuous variables will be reported as mean and standard deviation or median and interquartile range and will be compared using parametric tests (*t*-test, ANOVA) or non-parametric tests (Mann–Whitney test, Wilcoxon signed rank, McNemar tests) according to their distribution. Categorical variables will be reported as absolute number and percentage and will be compared using parametric tests if normally distributed (Chi-squared test, Fisher test) or non-parametric tests. Association between variables will be evaluated using correlation and regression analyses. Survival analysis will be performed on the basis of follow-up data (study Stages 2 and 3). We will conduct a Cox proportional hazards regression model examining the association between PTSD, HTN status, and their interaction effect on hazard of developing incident CVD. The presence of 4 main types of CVD (coronary heart disease, stroke, peripheral arterial disease and aortic disease) will be assessed.

In order to investigate the peculiarity of the group of patients with HTN and PTSD and to establish how the studied parameters affect the formation of the specified group, two methods of multicomponent analysis will be applied—factor analysis using the method of principal components and the method of logistic regression. The use of these methods will make it possible to identify a number of factors affecting the variability of indicators in the study groups, as well as to build a mathematical model for hypertension and PTSD. Statistical significance will be set at 2-sided *p* value < 0.05 for all analysis.

Power of the study: the expected number of enrolled subjects in Stage 1 of the project is about 500. Stage 2 will likely involve 200 subjects, based on the expected prevalence of PTSD and HTN. Stage 3 will involve 200 subjects for cardiovascular and hormonal assessment.

Based on the sample size formula [*S* = *Z*^2  ^× *P*^  ^× (1 − *P*)/*M*^2^, where *Z* score is 1.960 for 95% confidence level, *M*—the margin of error of 5% (0.05)], the number of respondents 384 is sufficient to obtain representative results. The group sizes were additionally rounded up to take into account the possibility of the appearance of distributions deviating from the normal distribution among the examined features. Group size analysis was performed using the “pwr” package in R using the *pwr.t.test* function. Parameters used: Type I error = 0.05, Power = 0.8.

Therefore, our stated sample size of 500 refugee women aged 18+ will allow us to obtain representative data.

The formation of a sample of 200 women at Stages 2 and 3 of the study was due to literature data on the 20%–40% prevalence of PTSD in refugees and data that PTSD occurs on average 2 times more often in women than in men.

A summary of the study according to SPIRIT and the list of assessments performed during the study are described in Supplementary Tables 1 and 2.

#### Research cooperation

Department of Psychiatry of the Medical University of Gdańsk (Gdańsk, Poland), Sopot Center for Integration and Support for Foreigners (Sopot, Poland), Cliniques Universitaires Saint-Luc Université catholique de Louvain (Brussels, Belgium), Faculty of Biology and Medicine (Lausanne, Switzerland), State Institution “National Scientific Center M.D. Strazhesko Institute of Cardiology, Clinical and Regenerative Medicine” of the National Academy of Medical Sciences of Ukraine (Kyiv, Ukraine), Government Institution “L. T. Malaya Therapy National Institute of the National Academy of Medical Sciences of Ukraine” (Kharkiv, Ukraine), Ivano-Frankivsk National Medical University (Ivano-Frankivsk, Ukraine).

## Strengths and limitations of this study

### Strengths

PTSD has been associated with HTN, particularly in military servants, war veterans and more recently in populations exposed to chronic violence. Refugees have particularly high prevalence of PTSD (up to 20%–60%). To our knowledge, the association between HTN and PTSD has not been investigated in refugees. This will be the first study to provide information on the relations between HTN and PTSD in a large group of women refugees.

State-of-the art measurement of office BP and 24-h ambulatory BP measurement in a subgroup.

This study may help designing a specific approach of cardiovascular risk management in subjects exposed to chronic violence, war, immigrants and refugees at risk for developing PTSD.

On this occasion, Ukrainian refugees will get access to medical care and psychological support in their native language.

### Limitations

During the study, a proportion of patients may return to Ukraine. However, as Poland hosted about 2 million Ukrainian refugees, it is unlikely that this will substantially affect enrolment in the study. Furthermore, thanks to local collaborations, information and follow-up of subjects returning to Ukraine will be obtained as much as possible, allowing a formal comparison with subjects remaining in Poland.

Since Ukrainian men aged 18–60 cannot leave the territory of Ukraine during the war, only women will be included in our study. However, while it is well known that PTSD is more frequent in women, its impact on HTN in populations exposed to war has not been studied specifically in this subgroup. Finally, the burden of cardiovascular diseases is typically underestimated in women, who are underdiagnosed and undertreated. Therefore, focusing on women is relevant both from a scientific and public health perspective.

## Discussion

The war in Ukraine has already caused several thousands of deaths and it will likely have long-term consequences on population health. It has been shown that one in five (22%) people who have experienced war or other conflict in the previous 10 years, will have depression, anxiety, post-traumatic stress disorder. In applying these estimates to Ukraine, the World Health Organization (WHO) expects that approximately 9.6 million people in Ukraine may suffer from a mental health condition (31). Trauma exposure at population level, as experienced currently in Ukraine, will likely have a huge impact not only on mental health and well-being but also on associated cardiovascular disease burden. Early management of Ukrainian refugees' health needs may substantially reduce this disease burden during an in the aftermath of the war. In particular, screening for and treating HTN and PTSD in this population may result in a large-scale reduction of cardiovascular morbidity and mortality in the long-term. Furthermore, such approach may be further tested and extrapolated to other populations exposed to war and chronic violence, migrants and refugees around the world.

*Medical benefits* of the project include better understanding and identification of pathological mechanisms underlying effects of war-induced stress on hypertension and cardiovascular disease in women, as well as clinical testing and implementation of diagnostic and therapeutic strategies (including targeted psychological support) that will help reducing cardiovascular risk in stress-exposed subjects. Focus on women is justified by the fact that: (i) women constitute the large majority of Ukrainian refugees; (ii) despite a higher prevalence of PTSD in women (ref) no specific data are available on the relation of HTN, cardiovascular diseases and PTSD in this subgroup; (iii) overall, the risk of cardiovascular diseases in women is underestimated partly because of the perception that women are “protected” by estrogen exposure (32).

*Social benefits* of the project are related to improved diagnosis of stress-induced hypertension and cardiovascular disease in women, provide an opportunity to provide them the necessary medical care outside their homeland in the native language. It will improve the social adaptation of women and their children who survived the war and as such may contribute to maintain quality of life and increase life expectancy in these women.

*Economic benefits* of the study will be determined by a significant increase in efficiency of diagnostic measures that will allow timely identification of war-induced stress and prevent the development and progression of hypertension and cardiovascular disease in women enrolled in the study, reduce treatment costs, and increase efficiency of treatment. In addition, it should facilitate the insertion of these women in the working environment.

### Complementary assessments

We plan to organize a *prospective assessment* of the state of the cardiovascular system and the severity of PTSD in women who remained on the territory of Poland since the beginning of the war in Ukraine compared with Ukrainian women who remained on the territory of Ukraine**.** Six months after enrolment in the first phase of the study, for those patients who, at the initial stage of the study, entered the extreme quartiles in terms of stress severity, an assessment of PTSD and trauma exposure will be provided and the research methods carried out in the stage 3 will be assigned. The results obtained will be compared with the results of the studied conditions in Ukrainian women who remained on the territory of Ukraine.

The consequences of war-induced stress are not only a major public health problem, but also a social problem that requires a complex solution. We have a mission to involve the wider scientific community, stakeholders and patient representatives at every stage of the project. This will ensure that significant progress is made and that data and tools from this initiative will be made available to other disease areas.

Our program of dissemination will ensure that this project has a long-term impact on lay public attitudes, patient expectations, and will generate new directions for integrated care across the health spectrum of war refugees and trauma victims.

## References

[1] Forouzanfar MH Liu P Roth GA Ng M Biryukov S Marczak L Global burden of hypertension and systolic blood pressure of at least 110 to 115 mm Hg, 1990–2015. JAMA. (2017) 317:165–82. 10.1001/jama.2016.19043 28097354

[2] Mills KT Stefanescu A He J. The global epidemiology of hypertension. Nat Rev Nephrol. (2020) 16:223–37. 10.1038/s41581-019-0244-2 32024986 PMC7998524

[3] Jula A Salminen JK Simo S. Alexithymia: a facet of essential hypertension. Hypertension. (1999) 33(4):1057–61. 10.1161/01.HYP.33.4.1057 10205248

[4] McRae K Gross JJ. Emotion regulation. Emotion. (2020) 20(1):1–9. 10.1037/emo0000703 31961170

[5] Kibler JL Joshi K Ma M. Hypertension in relation to posttraumatic stress disorder and depression in the US national comorbidity survey. Behav Med. (2009) 34(4):125–32. 10.3200/BMED.34.4.125-132 19064371

[6] Bapolisi A Maurage P Pappaccogli M Georges CMG Petit G Balola M Association between post-traumatic stress disorder and hypertension in Congolese exposed to violence: a case–control study. J Hypertens. (2022) 40(4):685–91. 10.1097/HJH.0000000000003061 34907991

[7] Edmondson D von Känel R. Post-traumatic stress disorder and cardiovascular disease. Lancet Psychiatry. (2017) 4(4):320–9. 10.1016/S2215-0366(16)30377-7 28109646 PMC5499153

[8] Mellman TA Brown DD Jenifer ES Hipolito MM Randall OS. Posttraumatic stress disorder and nocturnal blood pressure dipping in young adult African Americans. Psychosom Med. (2009) 71(6):627–30. 10.1097/PSY.0b013e3181a54341 19483123

[9] Sumner JA Kubzansky LD Elkind MS Roberts AL Agnew-Blais J Chen Q Trauma exposure and posttraumatic stress disorder symptoms predict onset of cardiovascular events in women. Circulation. (2015) 132(4):251–9. 10.1161/CIRCULATIONAHA.114.014492 26124186 PMC4519406

[10] O'Donnell CJ Schwartz Longacre L Cohen BE Fayad ZA Gillespie CF Liberzon I Posttraumatic stress disorder and cardiovascular disease: state of the science, knowledge gaps, and research opportunities. JAMA Cardiol. (2021) 6(10):1207–16. 10.1001/jamacardio.2021.2530 34259831

[11] Krantz DS Shank LM Goodie JL. Post-traumatic stress disorder (PTSD) as a systemic disorder: pathways to cardiovascular disease. Health Psychol. (2022) 41(10):651–62. 10.1037/hea0001127 34807673 PMC9124241

[12] Abouzeid M Kelsall HL Forbes AB Sim MR Creamer MC. Posttraumatic stress disorder and hypertension in Australian veterans of the 1991 gulf war. J Psychosom Res. (2012) 72:33–8. 10.1016/j.jpsychores.2011.08.002 22200520

[13] Burg MM Brandt C Buta E Schwartz J Bathulapalli H Dziura J Risk for incident hypertension associated with posttraumatic stress disorder in military veterans and the effect of posttraumatic stress disorder treatment. Psychosom Med. (2017) 79(2):181–8. 10.1097/PSY.0000000000000376 27490852 PMC5285494

[14] Howard JT Sosnov JA Janak JC Gundlapalli AV Pettey WB Walker LE Associations of initial injury severity and posttraumatic stress disorder diagnoses with long-term hypertension risk after combat injury. Hypertension. (2018) 71(5):824–32. 10.1161/HYPERTENSIONAHA.117.10496 29555664

[15] Jebril M Mazidi M Liu X Baibing M Arafat H Shi Z Association between war-related traumatic events and blood pressure trajectory: a population-based study among the mid-aged and older Palestinian adults living in gaza. Front Public Health. (2023) 11:1073284. 10.3389/fpubh.2023.1073284 37397782 PMC10310537

[16] Petit G Berra E Georges CMG Capron A Huang Q-F Lopez-Sublet M Impact of psychological profile on drug adherence and drug resistance in patients with apparently treatment-resistant hypertension. Blood Press. (2018) 27:358–67. 10.1080/08037051.2018.1476058 29952236

[17] Georges CMG Ritscher S Pappaccogli M Petit G Lopez-Sublet M Bapolisi A Psychological determinants of drug adherence and severity of hypertension in patients with resistant vs. controlled hypertension. Blood Press. (2022) 31(1):169–77. 10.1080/08037051.2022.2099346 35899361

[18] Persu A Petit G Georges C de Timary P. Hypertension, a posttraumatic stress disorder? Time to widen our perspective. Hypertension. (2018) 71(5):811–2. 10.1161/HYPERTENSIONAHA.118.10608 29555667

[19] Blackmore R Boyle JA Fazel M Ranasinha S Gray KM Fitzgerald G The prevalence of mental illness in refugees and asylum seekers: a systematic review and meta-analysis. PLoS Med. (2020) 17(9):e1003337. 10.1371/journal.pmed.1003337 32956381 PMC7505461

[20] PTSD: National Center for PTSD. Available at: https://www.ptsd.va.gov/ (accessed December 10, 2023).

[21] Operational data portal. Ukraine refugee situation. Available at: https://data.unhcr.org/en/situations/ukraine (accessed December 10, 2023).

[22] 2019 STEPS Country Report Ukraine. Available at: https://www.who.int/publications/m/item/2019-steps-country-report-ukraine (accessed December 10, 2023).

[23] Mancia G Kreutz R Brunström M Burnier M Grassi G Januszewicz A 2023 ESH guidelines for the management of arterial hypertension the task force for the management of arterial hypertension of the European society of hypertension endorsed by the international society of hypertension (ISH) and the European renal association (ERA). J Hypertens. (2023) 41(12):1874–2071. 10.1097/HJH.0000000000003480 37345492

[24] Stergiou GS Palatini P Parati G O’Brien E Januszewicz A Lurbe E European society of hypertension council and the European society of hypertension working group on blood pressure monitoring and cardiovascular variability. 2021 European society of hypertension practice guidelines for office and out-of-office blood pressure measurement. J Hypertens. (2021) 39(7):1293–302. 10.1097/HJH.0000000000002843 33710173

[25] Official DASS-21 test creator's. Available at: http://www2.psy.unsw.edu.au/dass/over.htm (accessed December 10, 2023).

[26] Peter J N. Depression anxiety and stress scales (DASS-21): psychometric analysis across four racial groups. Anxiety Stress Coping. (2007) 20(3):253–65. 10.1080/10615800701309279 17999228

[27] American Psychiatric Association and American Psychiatric Association. DSM-5 Task Force. Diagnostic and Statistical Manual of Mental Disorders: DSM-5. VA: American Psychiatric Association Arlington (2013).

[28] Weathers FW Litz BT Keane TM Palmieri PA Marx BP Schnurr PP. The PTSD checklist for DSM-5 (PCL-5). X: National Center for PTSD (2013). Available at: www.ptsd.va.gov (accessed January 04, 2024).

[29] García-Martín MA Hombrados-Mendieta I Gómez-Jacinto L. A multidimensional approach to social support: the questionnaire on the frequency of and satisfaction with social support (QFSSS). Anales de Psicología. (2016) 32(2):501–15. 10.6018/analesps.32.2.201941

[30] Graff G Graff B Pilarczyk P Jabłoński G Gąsecki D Narkiewicz K. Persistent homology as a new method of the assessment of heart rate variability. PLoS One. (2021) 16(7):e0253851. 10.1371/journal.pone.0253851 34292957 PMC8297888

[31] Scaling-up mental health and psychosocial services in war-affected regions: best practices from Ukraine. Available at: https://www.who.int/news-room/feature-stories/detail/scaling-up-mental-health-and-psychosocial-services-in-war-affected-regions–best-practices-from-ukraine (accessed January 04, 2024).

[32] Stramba-Badiale M Fox KM Priori SG Collins P Daly C Graham I Cardiovascular diseases in women: a statement from the policy conference of the European society of cardiology. Eur Heart J. (2006) 27(8):994–1005. 10.1093/eurheartj/ehi819 16522654

