# Supplementary material for: The impact of war on the development and progression of arterial hypertension and cardiovascular disease: protocol of a prospective study among Ukrainian female refugees
Source: Front Cardiovasc Med. 2024 Jan 11;10:1324367. doi: 10.3389/fcvm.2023.1324367 (PMC10808621; doi:10.3389/fcvm.2023.1324367)
Supplement: Supplementary file 1 [file Table1.docx]

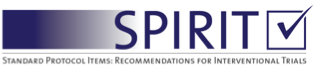


SPIRIT 2013 Checklist:

Recommended items to address in a clinical trial protocol and related documents*

Section/item Item No Description

**Administrative information**

**Title** 1 THE IMPACT OF WAR ON THE DEVELOPMENT AND PROGRESSION OF ARTERIAL HYPERTENSION AND CARDIOVASCULAR DISEASE: PROTOCOL OF A PROSPECTIVE STUDY AMONG UKRAINIAN FEMALE REFUGEES

Trial registration 2a Research study registration number 2022/45/P/NZ5/02812.

Protocol version 3 22.09.2022

Funding 4 Supported by the National Science Centre and the European Union’s Horizon 2020 research and innovation program under the Marie Skłodowska-Curie grant agreement No 945339.

Roles and responsibilities

5a Shalimova A., Department of Hypertension and Diabetology Medical University of Gdańsk, Gdańsk, Poland

Stoenoiu M.S., Department of Internal Medicine, Rheumatology, Cliniques Universitaires Saint-Luc, Université catholique de Louvain, Brussels, Belgium; Institut de Recherche Expérimentale et Clinique, Université catholique de Louvain, Brussels, Belgium

Cubała W.J., Department of Psychiatry, Faculty of Medicine, Medical University of Gdańsk, Gdańsk, Poland

Burnier M., Faculty of Biology and Medicine, University of Lausanne, Switzerland

Persu A. Cliniques Universitaires Saint-Luc, Université catholique de Louvain, Brussels, Belgium and Pole of Cardiovascular Research, Institut de Recherche Expérimentale et Clinique, Université catholique de Louvain, Brussels, Belgium.

5b None

5c None

5d Single center study.

I**ntroduction**

Background and rationale

6a

Growing evidence supports the impact of psychological factors such as traumatic experiences and Post Traumatic Stress Disorder (PTSD) on HTN and cardiovascular outcomes. This prospective study aims at the assessment of the impact of war-induced stress on HTN and cardiovascular disease (CVD) in women Ukrainian refugees who moved to Poland.

6b

The data collected in this study will be compared to cross-sectional data available from STEPS study (National study on the prevalence of major risk factors for noncommunicable diseases, in line with the WHO-approved phased approach to surveillance) and from May Measurement Month 2021 in Ukraine, matched for age and sex. Data from women remaining in Ukraine during the war time.

Objectives 7 To demonstrate that the prevalence of PTSD and hypertension are high in female refugees escaping war and that PTSD contributes to elevate BP and induce hypertension

Trial design 8 Single center, prospective open exploratory study

Methods: Participants, interventions, and outcomes

Study setting 9 Females refugees from Ukraine arrived in Gdansk region (PL)

Eligibility criteria 10 All women refugees are contacted and can accept or refused to participate. Subjects unable to understand and answer the questionnaires or patients with psychiatric disorders will be excluded.

Interventions 11a Women with an elevated blood pressure will be offered a standard treatment if their hypertension is confirmed, according to the standards of care in Poland. Patients with PTSD will be offered a psychological support.

11b Discontinuing the study will occur if patients refuse to continue.

11c No specific treatment is tested in this study.

11d None

Outcomes 12 Primary outcome: Prevalence of hypertension and PTSD at baseline

Secondary outcome: impact of PTSD on blood pressure, impact of PTSD with or without hypertension on cardiac, vascular, renal and metabolic phenotypes

Participant timeline 13 A figure is provided.

Sample size 14 500 subjects for stage 1, 200 subjects for stages 2 and 3

Recruitment 15 Direct contacts with refugees staying in dormitories or staying in the Gdansk area.

**Methods:** Assignment of interventions (for controlled trials)

Allocation:

Sequence generation 16a No randomization

Allocation concealment mechanism 16b None

Implementation 16c None

Blinding (masking) 17a None

17b Not relevant

**Data collection methods**

18a This research project has been approved by the Bioethics Committee for Scientific Research at the Medical University of Gdansk, Poland (reference number: NKBBN/558/2022). Participants will be recruited through promotion of this project in Sopot Centre for Integration and Support for Foreigners. A written informed consent in Ukrainian language will be obtained from all participants after the nature of the study is explained by an Ukrainian-speaking cardiologist. Participation will only be possible after written informed consent is provided.

The Stage 1 of our project will be organized in form of screening campaigns among Ukrainian refugees. Patients will be examined in an ambulatory environment by Ukrainian speaking investigators, thus providing them the possibility to access the Polish health system speaking in native language. Short past medical history information will be collected at this stage, including known history of hypertension (HTN), known HTN-associated organ damage, concomitant comorbidities and cardiovascular risk factors (i.e. smoking status, diabetes, renal disease, dyslipidemia), previous cardiovascular or cerebrovascular events, and past and current therapy.

Designated medical doctors will contact the patients by phone and invite them to the Translational Medicine Centre of the Medical University of Gdansk to explain the study, pass relevant information and if they agree, collect signed informed consent and schedule the study procedures.

At this stage, patients will have their office blood pressure measured and complete a stress questionnaire. Post-Traumatic Stress Disorder (PTSD) diagnosis will be confirmed on the basis of more advanced evaluation, i.e. Clinician-Administered PTSD Scale for DSM-5 (CAPS-5). To assess general emotional distress, we will use validated questionnaire DASS-21 (Depression, Anxiety and Stress Scale - 21). On the basis of this questionnaire, we may determine 5 levels of severity of the depression, stress and anxiety: normal, mild, moderate, severe and extremely severe.

At stage 2 of the project, it is planned to examine 200 patients (of those who will be examined at the first stage). At the request of the patients, persons with a confirmed diagnosis of PTSD will be referred to a psychiatrist or psychologist, followed according to standard of care and periodically reassessed for the severity of PTSD and emotional distress.

For Stage 3 of the project, voluntary subjects with HTN, PTSD, both or none of these two diagnoses will be selected for further evaluation of mechanisms mediating the association between HTN and PTSD. Information about lifestyle habits, such as smoking status, alcohol consumption, physical activity and dietary habits will be integrated/collected from data already gathered at Stage 1. Blood and urine samples will be collected in order to screen for other cardiovascular risk factors, such as dyslipidemia, fasting glucose intolerance and cardiac phenotype and diabetes, renal dysfunction, as well as to quantify sodium dietary intake.

18b The participation in the study will be voluntary, the study details will be explained to the potential participants by designated medical doctors, and they will have possibility to ask questions. Only after providing the potential participants with detailed information about the project, its protocol and possible risks, categories of personal data we will process and their rights under GDPR regulations, we will ask them to sign an informed written consent. We will provide them with the copy of the consent and associated ‘Patient Information Sheet’ containing all the information about the project and participants' rights, including the right to withdraw their consent at any time without giving a reason.

**Data management**

19 In our study we will comply with GDPR regulation and our internal Medical University of Gdansk Data Protection Policy for processing patient personal information, including special category data such as medical data. Lawful basis for processing patient personal data for our research study will be patient informed consent. Collected biological samples and associated data will be used only for the research purpose. All samples will be immediately pseudo-anonymized by giving them unique patient ID code. While this code provides a link to the donor's consent form and other identifiable donor information, this information will be stored separately and securely. To safeguard patient rights, we will use the minimum personally-identifiable information possible, necessary to access patient medical files and allow further contact (patient name, date of birth, hospital number, national ID number – PESEL, phone number and address). Only limited number of people who underwent internal GDPR training and received written permission to process personal data will have an access to the identifiable patients' information. The research staff, to whom the samples will be released in order to perform research experiments and analyze the data, will only have the access to the coded information.

All junior researchers will be trained in ethics and research integrity. Senior researchers will supervise, guide and train their team members to ensure research integrity and prevent research misconduct.

We will ensure appropriate management and curation of all data and research materials, with preservation of original.

**Statistical methods**

20a Statistical analysis will be performed using IBM SPSS Statistics Software v28.0.1.1for Windows (IBM Corp. Released 2021. IBM SPSS Statistics for Windows, Version 28.0. Armonk, NY: IBM Corp). Continuous variables will be reported as mean and standard deviation or median and interquartile range and will be compared using parametric tests (t-test, ANOVA) or non-parametric tests (Mann-Whitney test, Wilcoxon signed rank, McNemar tests) according to their distribution. Categorical variables will be reported as absolute number and percentage and will be compared using parametric tests if normally distributed (Chi-squared test, Fisher test) or non-parametric tests. Association between variables will be evaluated using correlation and regression analyses. Survival analysis will be performed on the basis of follow-up data (study Stages 2 and 3). We will conduct a Cox proportional hazards regression model examining the association between PTSD, HTN status, and their interaction effect on hazard of developing incident CVD. The presence of 4 main types of CVD (coronary heart disease, stroke, peripheral arterial disease and aortic disease) will be assessed.

Statistical significance will be set at 2-sided p value < 0.05 for all analysis.

20b In order to investigate the peculiarity of the group of patients with HTN and PTSD and to establish how the studied parameters affect the formation of the specified group, two methods of multicomponent analysis will be applied - factor analysis using the method of principal components and the method of logistic regression. The use of these methods will make it possible to identify a number of factors affecting the variability of indicators in the study groups, as well as to build a mathematical model for hypertension and PTSD.

20c Not relevant

**Methods: Monitoring**

Data monitoring 21a No data monitoring committee

21b Interim analyses will be performed after the first 100 subjects enrolled.

Harms 22 In the absence of studied interventions, one does not anticipated any harms. Aggravations of the subjects situation may be handled by regular medical care in the Gdansk hospital

Auditing 23 No audit will be performed

**Ethics and dissemination**

Research ethics approval 24 The protocol has been submitted and accepted by the Local Ethics Committee.

Protocol amendments 25 Any protocol modification or amendment will be submitted to the EC before being implemented.

Consent or assent 26a Informed consent will be obtained by the primary investigator (Dr A. Shalimova) who comes from Ukraine and speaks perfectly the subjects language. All documents have been translated in Ukrainian

26b None so far

Confidentiality 27 An anonymized database will be created to maintain the confidentiality

Declaration of interests 28 None

Access to data 29 Only members of the study group will have access to the data.

Ancillary and post-trial care 30 None

Dissemination policy 31a Data will be used for abstracts and scientific publications

31b No professional writers will be used

31c No plans so far in this direction.

**Appendices**

Informed consent materials 32 Model consent form and other related documentation given to participants and authorized surrogates

Biological specimens 33 Blood and urine samples will be collected in order to perform hormonal evaluation: renin, aldosterone, metanephrines, cortisol, C-reactive protein levels. Collected biological samples and associated data will be used only for the research purpose. All samples will be immediately pseudo-anonymized by giving them unique patient ID code. Biological material from patients (blood and urine) will be delivered to the laboratory immediately after blood collection for subsequent molecular analysis. Biobanking of biological material is not provided for in our study. Genetic analysis is not included in our study.

*It is strongly recommended that this checklist be read in conjunction with the SPIRIT 2013 Explanation & Elaboration for important clarification on the items. Amendments to the protocol should be tracked and dated. The SPIRIT checklist is copyrighted by the SPIRIT Group under the Creative Commons “Attribution-NonCommercial-NoDerivs 3.0 Unported” license.
