# Supplementary material for: The impact of war on the development and progression of arterial hypertension and cardiovascular disease: protocol of a prospective study among Ukrainian female refugees
Source: Front Cardiovasc Med. 2024 Jan 11;10:1324367. doi: 10.3389/fcvm.2023.1324367 (PMC10808621; doi:10.3389/fcvm.2023.1324367)
Supplement: Supplementary file 2 [file Table2.docx]

**Table 2. The list of assessments and interventions**

|  | **STUDY PERIODS** | | | | |  |
| --- | --- | --- | --- | --- | --- | --- |
|  | **Enrolment** | **Inclusion** | **Stage 1** | **Stage 2** | **Stage 3** | **Outcomes** |
| **TIME POINT** | -1 | 0 | 1 | 2 | 3 |  |
| **Eligibility screen** | X |  |  |  |  |  |
| **Informed consent** | X |  |  |  |  |  |
| **Inclusion** |  | X |  | X |  |  |
| **Assessments** |  |  |  |  |  |  |
| - Clinical and demographics |  |  | X |  |  |  |
| - BP measurements |  |  | X |  |  |  |
| - Stress assessment questionnaire |  |  | X |  |  |  |
| - Follow-up if willing |  |  |  | X |  |  |
| - Complete stress assessment |  |  |  | X |  |  |
| - Detailed assessment |  |  |  |  | X | X |
| CV risk factors |  |  |  |  | X |  |
| Hormones |  |  |  |  | X | X |
| Cardiac phenotype (ECG, Echo) |  |  |  |  | X |  |
| ABPM |  |  |  |  | X | X |
| Renal function |  |  |  |  | X | X |
| Metabolism (lipids, glucose) |  |  |  |  | X | X |
| Heart rate variability |  |  |  |  | X |  |
| PWV |  |  |  |  | X |  |
| **Interventions** |  |  |  |  |  |  |
| Treatment of hypertension |  |  |  | X |  | X |
| Psychological treatment of PTSD |  |  |  | X |  | X |
